# An Update on the Evolutionary History of Bregs

**DOI:** 10.3390/genes13050890

**Published:** 2022-05-17

**Authors:** Michel-Edwar Mickael, Irmina Bieńkowska, Mariusz Sacharczuk

**Affiliations:** 1 Institute of Genetics and Animal Biotechnology of the Polish Academy of Sciences, ul. Postepu 36A, Jastrzebiec, 05-552 Magdalenka, Poland; 2 PM Research Center, 20 Kaggeholm, Ekerö, 178 54 Stockholm, Sweden; i.bienkowska@igbzpan.pl (I.B.); m.sacharczuk@igbzpan.pl (M.S.)

**Keywords:** Bregs, evolution, adaptive immune system, regulation

## Abstract

The relationship between the evolutionary history and the differentiation of Bregs is still not clear. Bregs were demonstrated to possess a regulatory effect on B cells. Various subsets of Bregs have been identified including T2-MZP, MZ, B10, IL10-producing plasma cells, IL10 producing plasmablasts, immature IL10 producing B cells, TIM1, and Br1. It is known that B cells have evolved during fish emergence. However, the origin of Bregs is still not known. Three main models have been previously proposed to describe the origin of Bregs, the first known as single–single (SS) suggests that each type of Bregs subpopulation has emerged from a single pre-Breg type. The second model (single–multi) (SM) assumes that a single Bregs gave rise to multiple types of Bregs that in turn differentiated to other Breg subpopulations. In the third model (multi–multi) (MM), it is hypothesized that Bregs arise from the nearest B cell phenotype. The link between the differentiation of cells and the evolution of novel types of cells is known to follow one of three evolutionary patterns (i.e., homology, convergence, or concerted evolution). Another aspect that controls differentiation and evolution processes is the principle of optimization of energy, which suggests that an organism will always use the choice that requires less energy expenditure for survival. In this review, we investigate the evolution of Breg subsets. We studied the feasibility of Breg origination models based on evolution and energy constraints. In conclusion, our review indicates that Bregs are likely to have evolved under a combination of SM–MM models. This combination ensured successful survival in harsh conditions by following the least costly differentiation pathway, as well as adapting to changing environmental conditions.

## 1. Introduction

Exploring the contribution of Bregs to the field of autoimmunity and tolerance is gaining momentum [1,2,3]. In both autoimmune diseases and cancer, regulation of the immune system is important. Tregs and Bregs are the two central cells controlling the regulation of the adaptive immune system [4,5,6,7]. The capability of a B cell subpopulation to induce tolerance was first described by Katz et al. In their research, the authors proved that depleting B cells from spleen cells resulted in eliminating the ability of splenocytes to inhibit inflammatory response [8]. Later, it was demonstrated that mice deficient in B cells showed a different experimental autoimmune encephalomyelitis onset and severity, and did not demonstrate spontaneous remission compared to controls [9]. Subsequently, it was discovered that a specific B cell subpopulation termed “Bregs” is capable of producing IL10 and TGFβ leading to inflammation reduction [10,11]. Currently, Bregs are widely investigated in order to understand their crucial role in the regulation of the adaptive immune system.

The evolutionary history of Bregs is currently fragmented. Pre-B cells and plasma cells have been reported in the earliest jawed vertebrates, namely fish and sharks [12]. However, there currently has not been any considerable effort to localize Breg populations in these species. As a subpopulation of B cells, various attempts have been made to decipher the position of Breg differentiation within the B cells development map. Various models infer and discuss the origin of Bregs [13]. However, none of these models take into account the evolutionary history of Bregs and B cells. There are various known models that aim to explain the link between novel cell emergence and their differentiation [14]. However, using these evolutionary models has not been yet applied to understand Bregs’ evolutionary history.

In this review, we aim to shed light on the evolutionary relationship between various Breg subpopulations in relation to B cell differentiation. For that, we summarize various cellular evolution models that could solve the riddle of Breg emergence (e.g., homology, convergence, and concerted evolution). We summarize what is currently known about B cell differentiation in three main models (humans, rodents, and fish). We particularly have chosen these three organisms as most of the discoveries and characterization of B cells have been made in rodents, and due to the fact that fish represent the earliest diverging branch of organism that possess a B cell receptor (BCR). BCRs are the main structure with which B cells recognize antigens and it is the hallmark of B cells. Using this knowledge, we deduce the B cell’s evolutionary pattern. After that, we summarize Breg group marker evolution and its relationship to cellular differentiation. Following that, we combine the models of B cell differentiation–evolution modes with the evolution models of Breg emergence to examine their feasibility.

## 2. Evolution of Cell Types

**Figure 1:**
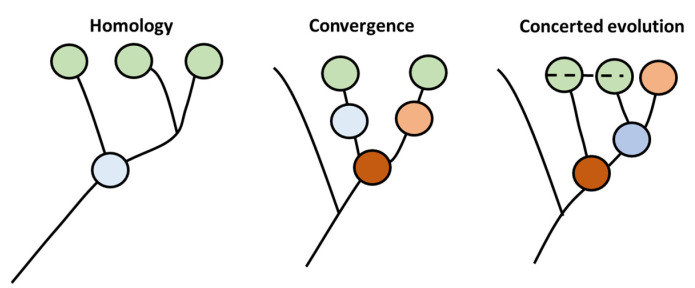
CoRc evolution produces novel cell types adapted from [14]. Three types of evolution patterns have been hypothesized to produce cellular diversity, namely homology, convergence, and concerted evolution. Similar color represents similar phenotype. The dotted line represent exchange of genetic material.

One of the primary aspects of evolution is the production of new species. This step is fundamentally based on the ability of evolutionary patterns to generate cellular diversity [14]. This pattern is governed by the cell type-specific core regulatory complex (CoRC) genes. This complex is composed of genes that are responsible for diversification of new cells types by controlling the transcription of groups of genes that would be responsible for novel emerging morphology. Currently, more than one mechanism have been proposed to envisage the role of evolution in the diversification of cellular types, and especially in the generation of CoRC (Figure 1). In the first model, known as the homology model, the new cellular types are generated through production of new offspring that are highly similar in morphology to precursor cells based on sharing a common ancestor precursor cell. In the second type, known as the convergent model, novel types emerge without sharing a common ancestor with their precursors. In that case, similarity in morphology is based on similarity in adaptions to environmental conditions rather than on high degree homology. The third type is called concerted model. In this type of evolution, genes from different cells within the same organism coordinate their evolution, possibly caused by the exchange of genetic material in an analog way to multicopy rDNA evolution in animals.

Another aspect that contributes to controlling the evolution process is the principle of optimization of energy. This principle is based on the observation that gathering energy in a dangerous environment is costly. Thus, acquiring energy is expensive. Hence, given the choice, a biological system will self-regulate to choose the least costly option needed for survival.

## 3. B Cell Differentiation and Evolutionary History

In this section, we cover the main groups of B cells, their differentiation patterns, and their evolutionary patterns. In the next section, we discuss Breg subpopulations and deduce their evolutionary history. After that, based on this information, we investigate the feasibility of Breg evolution models in light of current Breg differentiation models.

### 3.1. B Cell Main Groups

B cells can be grouped according to their function. Pro-B cells’ main function is lineage commitment. Pre-B cells express a pre-B cell receptor (pre-BCR). Pre-BCR is formed of μ proteins in association with two other proteins that resemble light chains and are thus called surrogate light chains (SLC). Pre-BCR promotes the survival and the proliferation of B cells by delivering signals to B cells that have made successful rearrangements of their Ig heavy chains [15]. Pre-BCR also inhibits further recombination of Ig heavy chain genes to ensure allelic exclusion [16]. Moreover, pre-BCR also induces the recombination of the K-type light chain. After the production of the light chain, pre-BCR delivers survival signals only for cells that expressed the complete antigen receptor. Immature B cells are the first B-lymphocyte that express B-cell receptors (BCR) on the cell surface. Immature B cells undergo several processes to eliminate self-reactive B cells such as deletion, receptor editing, and anergy [17]. In mammals, transitional B cells represent the link between immature B cells found in the bone marrow, and the naïve mature B cells found in the spleen. Naïve B cells express two classes of membrane-bound antibodies, namely IgM and IgD [18]. These two membrane-bound antibodies act as receptors for antigens. Activation of the naïve B cells leads to clonal expansion. Furthermore, it was demonstrated that follicular B cells reside in the follicles of the lymphoid organs in mammals. Follicular B cells are critical for mounting the T cell-dependent response, as well as the production of highly specific antibodies. They can also give rise to plasma cells. Marginal-zone B cells seem to be restricted to the peripheral region of the white pulp in the spleen [19]. One of their main functions is responding to polysaccharide antigens. Plasmablasts derived from follicular B cells or marginal zone B cells normally migrate to the bone marrow and mucosal tissues, where they differentiate into long-lived plasma cells.

### 3.2. B Cell Differentiation

**Figure 2:**
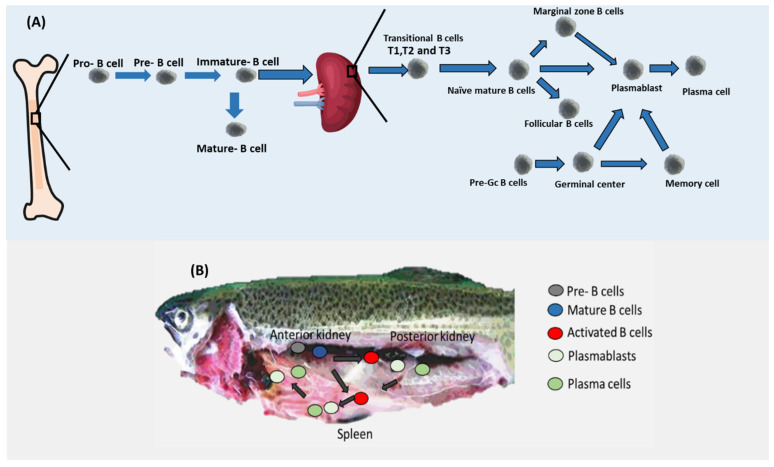
B cell differentiation. (**A**) After appearing in the bone marrow, pre-B cells are transferred to pro-B cells which in turn are converted into immature B cells. Immature B cells leave the bone marrow and migrate to the spleen in the form of transitional B cells that are converted into naïve mature B cells. Naïve mature B cells have three possible fates, namely marginal zone B cells, follicular B cells, and plasmablasts. Pre-Gc cells can also be converted into plasmablasts. Plasmablasts can be transferred into plasma cells. Currently, the position of Bregs within this traditional B cell differentiation map is still obscure. (**B**) B cells in fish. Whereas pre cells were found in the anterior kidney regions in trout, activated B cells seem to migrate to the posterior kidney region and the spleen where they differentiate into plasmablasts and plasma cells.

In mammals, B cell development commences in the liver of the fetus and continues to develop in the bone marrow [20,21,22]. Various stages of B cell development are distinguishable by combinations of cell surface and intracellular markers, cell cycle profile, and the rearrangement status of IgH and IgL genes. B cell commitment is determined by the transcription factor Pax5, one of whose target genes is CD19 [22,23]. The bone marrow CLP/pre-pro-B cell is the earliest B cell progenitor, and it was shown that it expresses B220 and has germline Ig genes [24,25]. After that, human pro-B cells lose their CD34 marker to become pre-B cells (Figure 2) [26]. In the case of mice, pro-B cells lose their CD43 marker to become pre-B cells. It is important to note that pre-B cells are defined by the expression of Igμ heavy chain protein in the cytoplasm. Following that, in both humans and mice, pre-B cells are converted into immature B cells. Immature B cells are different from pre-B cells in carrying the marker IgM [27,28]. It was reported that 3% of immature B cells can be converted to mature B cells. On the other hand, most of the immature B cells produced in the bone marrow leave the bone marrow as transitional cells by gaining the maker CD24 in both mice and humans [29]. Interestingly, transitional B cells can exist in three subtypes known as T1, T2, and T3 in mice, whereas this proposed heterogeneity is not yet clear in humans. Naïve mature B cells differentiate from transitional B cells. Naïve mature B cells can be converted into Follicular B cells or marginal zone B. Both marginal zone B cells and follicular B cells, as well as naïve mature B cells, can be converted into plasmablasts. Plasmablasts in turn can be transferred to plasma cells in the case of humans. It was also shown that pre-GC B cells that appear in the spleen with their markers of CD19+ CD38+ IgD+ can be converted to germinal center cells. Germinal center B cells can be converted into either plasmablasts or memory C cells (CD19+ CD27+).

B cell development in the anterior kidney of fish has been experimentally validated (Figure 2). Several genes have been proposed to support B development in Actinopterygii (ray-finned fishes) such as RAG-1/2, TdT, and transcription factor Ikaros [12]. Additionally, CD22 and CD79A seem to mark most of the B cells in salmon [30]. In rainbow trout (Oncorhynchus mykiss), mature B cells including both resting and activated B cells seem to reside in the anterior kidney. Similar to mammals, fish mature B cells’ main marker was reported to be Pax5. Pax5 was shown to inhibit the production of Igs. In the case of the presence of antigens, mature resting B cells become activated and increase the level of expression of MHC II while decreasing the level of Ig. Once activated, B cells migrate to the posterior kidney region and the spleen where they differentiate into plasmablasts by increasing the expression of Blimp-1, which is known to inhibit the production of Pax5 resulting in upregulation of production of Igs [12,31,32]. Interestingly, not all B cell types have been reported in fish. For example, T1, T2, MZP, naïve mature B cells, and Follicular B cells have not been yet demonstrated in fish (Table 1).

### 3.3. Evolution of B Cells

**Figure 3:**
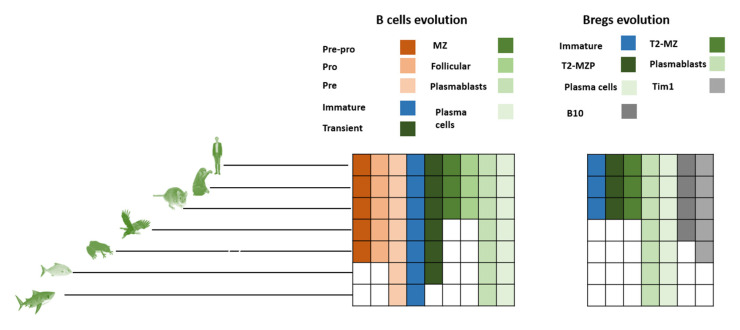
B cell evolution versus Breg evolution. Pre-B cells, immature B cells as well as plasma cells and plasmablasts have been found in lower vertebrates. However, fish and sharks lack the regions needed for the existence of follicular B cells. Our phylogenetic investigation demonstrates that only plasmablasts, Bregs, and plasmablast B cells exist in fish (bony fish and cartilaginous fish).

**Figure 4:**
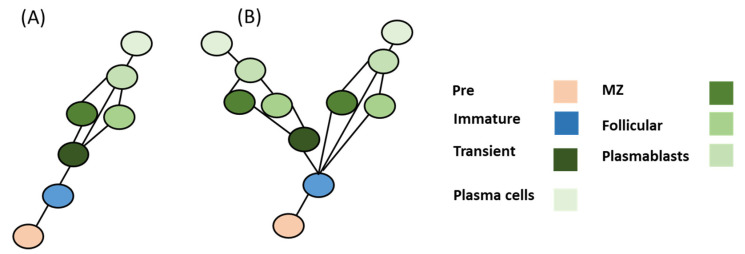
Evolutionary model of B cell differentiation. (**A**) Model A offers a simple homology of a pattern with one unresolved node based on the assumption that transitional B cells were to be localized in sharks. (**B**) Model B assumes that no transitional B cells were to be found in the sharks. This model suggests that MZ and follicular B cells have risen from a convergent or concerted evolution pattern.

B cells seem to have first emerged in early fish species. Invertebrates possess a few components of the basic requirements of the B cell phenotype, such as cytokines IL14, IL41, and IL17, as well as several cytokine receptors such as IL17RA, IL17RD, and gp130 [35]. However, most of the genes required for selective recognition of antigens for the BCR systems seem to be missing [36,37]. One of the fundamental genes responsible for the BCR system, namely Rag1/2, is expressed in Tunicates. However, Tunicates lack any apparent adaptive immunity system, suggesting that Rag had a separate ancient role that evolved in vertebrates to produce BCR diversity. Jawless vertebrates do not possess Rag1/2 or BCRs. However, they have a homologous system of antigen recognition known as the VLR system. The VLR system consists of three primary cells; VLRA, VLRB, and VLRC. VLRC seems to be the nearest analog for B cells, as they release VLRB molecules that are antigen specific. Jawless vertebrates do not possess lymph nodes. VLRB seems to develop in the kidney or an invagination in the intestinal epithelium. Fish (cartilaginous and bony) are the first vertebrates that possess the necessary mechanism for BCR as well as MHCII. Bony fish possess B cells that can be classified as plasmablasts and plasma cells. However, they do not possess bone marrow. The fish kidney and spleen are the major areas where B cell development takes place. Currently, no pre-pro-B cells have been experimentally identified. The existence of pre-B cells has been validated in bony fish. Immature B cells have been found in sharks as well as bony fish [38]. Transitional cells have been shown to exist in fish [39,40]. However, transitional B cells have not been yet localized in cartilaginous fish. Cartilaginous fish, as well as bony fish, seem to lack follicular areas for follicular B cell development. Similarly, MZ B cells have not been yet found in either bony or cartilaginous fish (Figure 3). Since follicular B cells are lacking in fish, the evolutionary history therefore had at least one more instant of convergent evolution. Additionally in this case, no transitional B cells would be found in cartilaginous fish, that might mean an additional convergent node in B cell evolution (Figure 4).

Thus, there now exists two evolutionary scenarios. Model one assumes that there are transitional B cells in cartilaginous fish (e.g., sharks). In this model (Model A), it is assumed that model duplication and mutation of genes resulted in the appearance of novel CoRCs that were able to modify terminal genes responsible for forming B cell phenotypes, albeit with one unresolved node (e.g., MZ or follicular cells). In this model, B cells are assumed to have followed a homology evolution pattern that closely followed the differentiation process except at the follicular MZ differentiation nodes (Figure 4). Model B assumes that sharks did not evolve to have these transient B cell phenotypes. It could be feasible that activated B cells can differentiate directly toward plasmablasts in cartilaginous fish. In that case, the MZ and follicular B cells could have risen from a convergent evolution or concerted evolution patterns.

## 4. Breg Evolutionary History

In this section, we discuss the main populations of Bregs, markers, evolution, and models of differentiation and investigate the feasibility of evolutionary models based on available Breg differentiation theories.

### 4.1. Breg Subpopulations

Bregs are known for producing IL10 and exerting a suppressive function similar to that of Tregs. The anti-inflammatory role of Bregs was demonstrated in various diseases. It was shown that mice lacking IL10 producing B cells displayed a pro-inflammatory Th1 response and were not able to recover after EAE induction [9,41]. Furthermore, it was reported that mice lacking Bregs suffered from increased arthritis along with an increase in Th1 and Th17 numbers. Although eight Bregs populations have been reported, the difference between their functions among different species is not well documented. Of these eight subpopulations, only four have been experimentally found in humans, namely plasmablasts, plasma cells, Br1, and B10 Bregs (Table 2). **Plasmablast Bregs in mice** seem to develop outside the spleen possibly in the draining lymph node. Their primary function is to prevent antigen-presenting cells from activating pathogenic T cells through the production of IL10. This function was demonstrated by using IL-10 reporter mice that were subjected to EAE induction. Mice lacking these regulatory plasmablasts demonstrated a higher degree of inflammation [42]. Plasmablasts were confirmed to exist in humans and perform a similar function to that of mice [42]. B10 also exists in humans and mice and produces IL10. B10 is predominantly expressed in the spleen and they are phenotypically different from plasmablasts [43]. Notably, the origin of B10 cells is not yet known [43]. Immature Bregs have only been demonstrated so far in humans. It was shown that, after CD40 stimulation, immature Bregs suppressed Th1 1 cells through the production of IL10. Moreover, immature Bregs isolated from systemic lupus erythematosus patients were resistant to CD40 stimulation and produced lower amounts of IL10 [44]. **Plasma cell Bregs** have only been reported in **mice** and they seem to follow the line of the Breg suppression of immune cells in EAE and salmonella infection by producing IL10. Additionally, they were shown to produce IL35 which plays an important role in immune cell suppression downregulating the B cells’ antigen presentation capabilities. Importantly, their function is mediated by Myd88 [45,46]. T2-MZP Bregs are also IL10 producing cells and they are mainly resident in the spleen [47]. T2-MZP cells seem to have been experimentally demonstrated in mice but not in humans. T2-MZP functions by producing IL10 and has been shown to contribute to the prevention of arthritis [48]. It seems to become activated following pathogen interaction through the TLR pathway. They are then further activated through CD40, BCR, and CD80-CD86 pathways increasing IL10 production. Interestingly, it has been recently shown that they were upregulated in response to the infection caused by the nematode parasites Trichinella spiralis in mice [10]. Moreover, they were shown to produce IL10 and express Hes1 and Deltex1 [49,50]. The main descendants of T2-MZP cells are MZ B cells. MZ B cells have been reported to suppress antigen-specific CD8+ T-cell responses during the early stages of Leishmania donovani infection [13]. MZ cells express polyreactive BCRs, thus they can bind to multiple microbial molecular patterns [51]. Activating signals for MZ cells in mice include Capsular polysaccharides, TLR ligands, BAFF, APRIL, CD40L, complement proteins, and IFNγ [51]. Another mouse specific Breg is B1a. This peculiar Breg population is derived from non-conventional B2 cells. B1A are innate immune cells that are capable of producing IgM [52].

### 4.2. Evolution of Bregs

#### 4.2.1. Immature Bregs

Immature Bregs seem to have emerged during the period of emergence of rodents. Immature Bregs are marked by CD19, CD24, and CD38. Notably, although CD38 diverged during lamprey emergence, CD19 and CD24 diverged much later (in amphibians and rodents, respectively). Critically, the emergence of immature Bregs seems to have occurred after the emergence of immature B cells in fish, possibly in rodents. However, this can be disputed based on the usage of CD19 and CD24 as markers for pre-B cells. Interestingly pre-B cells have been reported in fish that lack both markers (Table 1) [12]. Thus, the role of CD19 and CD24 as ultimatum markers of immature Bregs is not conclusive and makes it impossible to pinpoint the exact time of the emergence of immature Bregs.

#### 4.2.2. T2-MZP Bregs

The earliest evidence of T2-MZP is in rodents. The main markers of T2-MZP are CD21, CD23, CD1D, IgM, CD19, CD93, Dletex1, Hes1, Notch2, and CD24. Interestingly, all these markers are equally expressed in mice and humans, thus indicating that T2-MZP could also exist in humans. Interestingly, CD24 seems to be missing from Vombatus, Platypus chicken, Xenopus, and fish. CD24 is expressed on hematopoietic cells, including B cells. It is shared as a marker in four different Bregs (T2-MZP, B10 (humans), plasmablasts, and immature cells). Not expressing CD24 might indicate that T2-MZP, B10, and plasmablasts only started to emerge in rodents. It could also indicate that T2-MZP cells in lower vertebrates lack the functions mediated by CD24.

#### 4.2.3. MZ Bregs

Another type of cells that seems to have diverged in mice is MZ cells. MZ cells seem to produce IL10, induce Tregs, and suppress effector T cells. The main markers for these interesting cells are BAFF, APRIL, CD40L, and IFNγ. They seem to share CD19 and CD21 with the TZ-MZP cells. All defined markers seem to be expressed in both mice and humans, thus indicating that these cells could be found in humans. They are equally expressed in Vombatus indicating that MZ cells could have diverged before T2-MZP. Interestingly, CD40L has not been found in platypuses. This observation indicates that MZ cells could be lacking in platypuses or the that the divergence date lies after platypus emergence. Looking further back to Sauropsida, we observed that all indicated markers exist in Sauropsida, pushing back the time of divergence to at least 320 million years ago. CD21 seems to be missing in Xenopus, but not from bony fish or cartilaginous fish. CD19 seems to be missing from both bony and cartilaginous fish. Lampreys only contain APRIL and BAFF homologs, while Tunicates only include CD40L. Taken together, these observations indicate that MZ cells diverged during the time of the emergence of Sauropsida.

#### 4.2.4. Plasma Bregs

In agreement with plasma cells, plasma cell Bregs first diverged in sharks. The main markers of this group of cells are CD138 (SDC1), MyD88, and B220 (PTPRC). These cells were shown to have been long-lived plasma cells as well as having high expressions of IgM. It was found that IL10 production is strongly correlated with the expression level of Prdm1 (encoding the Blimp-1 protein), which is known to regulate plasma cell development [55]. Interestingly, all markers of plasma Bregs are expressed in earlier vertebrates including bony fish and sharks. Conversely, lampreys do not express CD138, although they express the rest of the investigated markers.

#### 4.2.5. Plasmablast Bregs

Plasmablast Breg divergence seems to have followed that of plasmablasts. Plasmablast Bregs (mice) have not been demonstrated in humans. Their primary markers are CD138 and CD44. Interestingly, both markers appear in animals as ancient as sharks/lampreys. In the case of human plasmablasts, their markers are CD19, CD24, IRF4, and CD27. It could be noticed that CD24 and IRF4 both diverged during the divergence time of rodents. This puts human plasmablast emergence at a later date in compared to plasmablasts from mice.

#### 4.2.6. B10 Bregs

Our investigation indicates that B10 emerged during Sauropsida (e.g., birds) divergence. There are two groups of B10. B10 mouse has been described predominantly in mice. Their primary markers are CD5 and CD1dhi, as well as CD19 and IL21 [56,57]. While in humans its two main markers are CD24hi and CD27+. Notably, B10 mouse markers seem to have been expressed in higher primates including humans. Whether or not B10 in mice and B10 in humans are mutually exclusive is still not known. B10 markers including CD19, CD1d, IL21, and CD5 are expressed as far as back as the time of Sauropsida. On the other hand, CD24 which is a marker for B10 in humans is only expressed in higher vertebrates, and it seems to be lacking in Vombatus and organisms that diverged before it. Thus, this puts the divergence of this group around the time of the divergence of B10 around 100 million years ago.

#### 4.2.7. Tim1 Bregs, BR1, and Breg (IL33)

**Figure 5:**
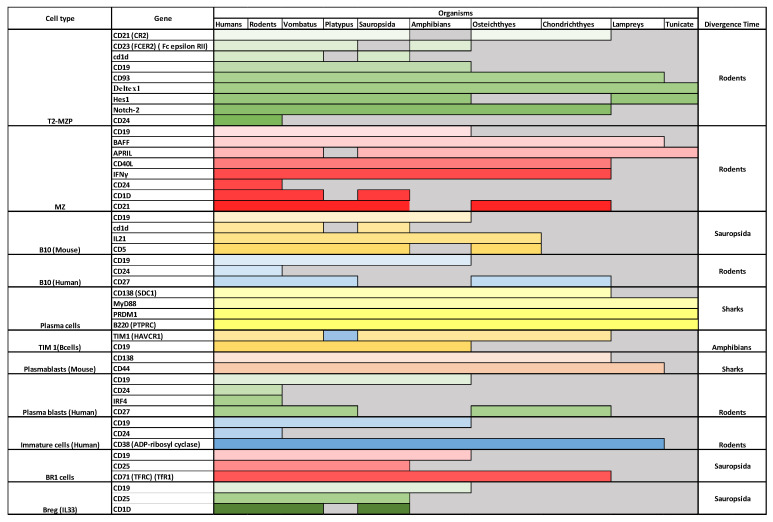
**Evolution of known Breg markers**. B cell emergence seems to have predominantly emerged during fish divergence. In contrast, Bregs’ current markers indicate a continuum of divergence. Each type of cell is represented by a different color shade. To produce these results, we followed a classical phylogenetic approach by downloading human proteins from NCBI (last access 5 of May 2022) and then blasting them against rodents, Vombatus, Platypus, Sauropsida, Amphibians, Osteichthyes (e.g., bony fish), Chondrichthyes (e.g., cartilaginous fish), Lamprey and Tunicate [35,60,61,62].

Tim1 Bregs seem to have diverged during amphibian emergence (Figure 5). Tim1 Bregs are marked by the expression of TIM1 as well as CD19 [58,59]. T cell Ig domain and mucin domain protein 1 (TIM-1) is a costimulatory molecule that regulates immune responses by modulating CD4+ T cell effector differentiation. It was shown that a subpopulation of Bregs expresses TIM1. It seems that Tim1 plays a role in the reciprocal regulation of B and T cells and the production of IL10. As described earlier, CD19 seems to be missing in both bony and cartilaginous fish, which moves the putative emergence of Breg Tim1 cells into the period of the emergence of amphibians during the Devonian period around 420 million years ago. Furthermore, BR1 and Breg (IL33) could not have had diverged before the emergence of Sauropsida.

### 4.3. Breg Development Models

**Figure 6:**
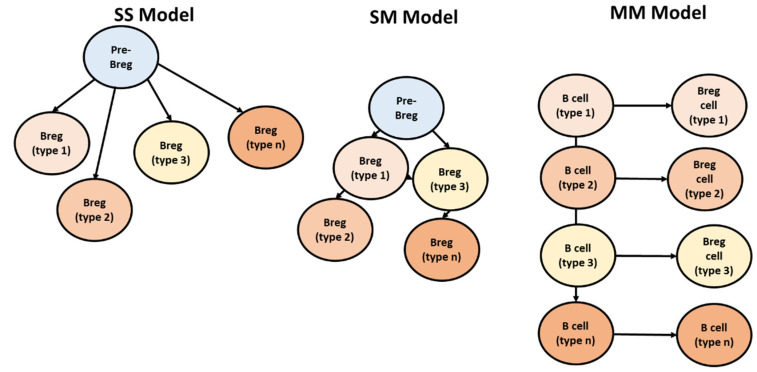
Three models have been suggested for Breg differentiation, adapted from [13]. In single–single model; each Breg type arises from a single Bregs progenitor. In single–multi model; one pre-Bregs gives rise to multiple Bregs. In the multi-multi, environmental conditions induce differentiation of Bregs from the nearest B cell homolog.

Multiple models have tried to address the origination and lineage of Bregs. (i) Model single–single (SS). In this model, it had been postulated that Bregs could have risen from an individual precursor/progenitor (Figure 6). This single progenitor can differentiate to any of the Bregs based on environment. (ii) In the second model (i.e., single–multi (SM)) various ancient populations of Bregs arise from a single Bregs progenitor, and these ancient populations give rise to novel types. (iii) Model multi–multi (MM); in this model, any B cell can become regulatory on exposure to specific environmental stimuli and exhibit suppressive capacity [13].

### 4.4. Deduction of Breg Evolution Models

**Figure 7:**
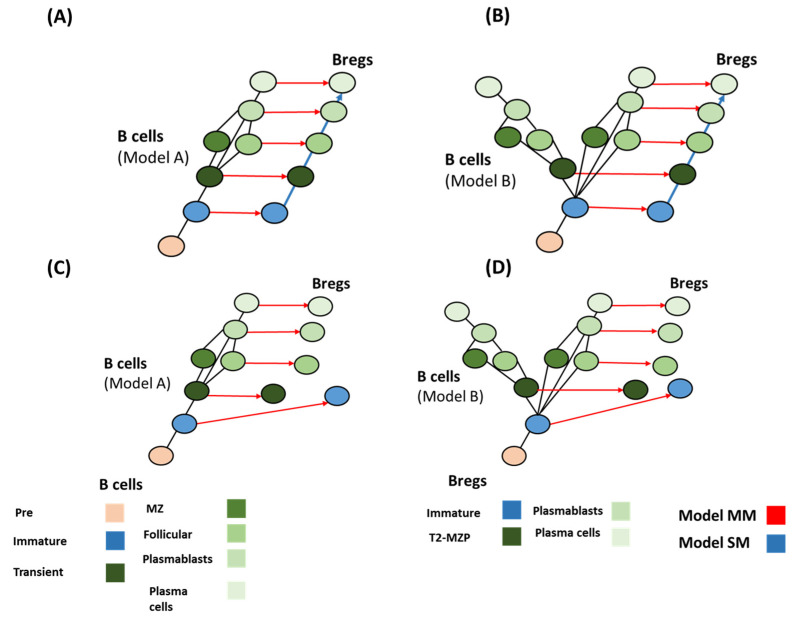
Possible mechanisms of Breg emergence. (**A**,**B**) Model MM and model SM best fit the data for both evolutionary models of B cells (model A and model B), in the case of immature Bregs being found in fish. Conversely (**C**,**D**), in the case that immature Bregs are missing in fish, model MM and SS best fit the data. These different combinations do not reveal the origin of B10 and Tim1.

Using the combination of differentiation and evolution models could lead to forming a hypothesis for identifying the evolutionary pattern of Bregs. Bregs’ evolution did not follow a simple homology pathway (Figure 5). There are two factors that regulate the logical deduction process: (a) the possibility of localizing transitional B cell populations in sharks (Figure 3); and (b) the possibility of finding immature Bregs in sharks (Figure 5). Based on these two assumptions, four main scenarios could be formed. (i) If Transitional B cells are found in sharks (model A), and immature Bregs are found in sharks, the best models that fit this assumption are MM and SM. Both models seem to fit with a homology-based evolution. Model SS does not follow the homology principle or convergence or concerted evolution (Table 3). (ii) Assuming model B is more likely, it would not result in changing the evolutionary patterns (Figure 6). (iii) In the case of model A and under the condition that immature Bregs are not found in sharks, model MS and SM do not fit the homology pattern alike, while model MM best fits this pattern. (iv) The differentiation, model MM is also the best fit under the differentiation model B and under the assumption that immature Bregs are not to be localized in sharks (Figure 7). Notably, these combinations do not resolve the origin of B10 or Tim1. This observation suggests that these two cells have convergent or concerted evolution patterns that will become clearer as further advancement in the understanding of the evolution of Bregs in fish takes place.

Taken together, these observations indicate that the most likely evolutionary model of Breg evolution is a combination of MM and SM models. Our review excludes the SS model as a reliable model for Bregs evolution as it does not agree with any of the evolutionary differentiation patterns currently known. Another base for exclusion of the SS model is the large cost burdened by the cell to evolve into a distant phenotype when an adjacent phenotype is available. Conversely, according to the MM–SS model a single Breg cell has first evolved in early cartilaginous fish (e.g., sharks) as a regulatory mechanism to restrict the function of inflammatory cells through a genetic mutation that leads to the overproduction of IL10. This single cell has evolved and differentiated according to the least costly pattern, presumably following the homology evolution mechanism (under an SM pattern). In some instances, however, the environment played a role in inducing novel phenotypes (under MM). The cell switching between the two methods is based on the optimization of the cost principle. In this form, the cell ensures survival in harsh conditions.

## 5. Conclusions

This Breg evolution study paints a complex evolutionary history of B cell evolution. Pre-B cells, as well as plasmablasts and plasma cells, have been found in fish. However, various other populations including follicular B cells, marginal zone, and T1 and T2 cell types are yet to be found. Anatomical constraints seem to have restricted the evolution of B cells and Bregs in early vertebrates, thus limiting the possibility of localizing some of these populations such as follicular B cells homologs. However, there are no currently known arguments against the possible localization of other populations, such as transitional B cells in cartilaginous fish. Investigating the evolution–differentiation relationship between Bregs and B cells showed that Breg emergence is likely the output of a homology evolutionary process that took place under an SM–MM differentiation model. It is also worth noting that other Breg cell types such as B10 and Tim1 do not follow the evolutionary models suggested here. Thus, they are more likely to have evolved through convergence or concerted evolution. Overall, future fish comparative immunology studies are needed to shed more light on the origin and evolution of Bregs.

**Authors Contributions:** M.-E.M. and M.S. conceptualized the paper. M.-E.M. and I.B. contributed to writing, M.S. supervised the writing and edited the manuscript. All authors have read and agreed to the published version of the manuscript.

## Figures and Tables

**Table 1:** Various B cells in comparison with literature findings in fish.

Cell Type	Humans	Mice	Fish
B cell commitment	Pax5	Pax5	Pax5
Pre-pro	CD117 CD10 CD34^+^ CD38^+^ Pax5^+^	Lin– B220/CD45 R^+^ CD19^−^ CD24low CD43^+^ C1q R1/CD93^+^ CD117/c-kit^−^ CXCR4^+^ Flt-3/Flk-2^+^ IL-7 Ra^+^ IgM	Not reported in fish
Pro-B cells	(CD19^+^ CD10^+^ CD34^+^ IgM^−^)	Lin– B220/CD45R^+^ CD19^+^ CD24^+^ CD43^+^ CD117^low^ IL7 Rα^+^ IgM^−^	Not reported in fish
Pre-B cells	(CD19^+^ CD10^+^ CD34^−^ IgM^−^)	Lin^−^ B220/CD45R^+^ CD19^+^ CD24^+^ CD43^−^ IL-7Rα^+^ IgM^−^.	B220 [12]
Immature B cells	CD10^+^ CD19^+^ CD20^+^ CD21^+^ CD24^+^ CD27^−^ CD38^+^ CD40^+^ CD93^+^ IL4Rα^+^ IL7 Rα^−^ [33]	CD45R^+^ CD19^+^ CD24^+^ CD43^−^ CD93^+^ IgD^−^ IgM^+^	
Transitional cells	CD10^+^ CD5^+^ CD19^+^ CD20^+^ CD21^+^ CD23^+^ CD24^+^ CD27^−^ CD38^+^ CD93^+^ TACI^+^	CD19^+^ CD10^+^ CD34^+^ CD24^+^ IgM^+^	Not reported in fish
Naïve mature B cells	CD19^+^ CD38^+^ and IgD^+^ [21]		pax5 [12,34]
Follicular B cells	CD10^−^ CD19^+^ CD20^+^ CD21^+^ CD22^+^ CD23^+^ CD24^low^ CD27^−^ CD38^low^ CXCR5^+^ TACI^+^.	CD45R+ CD1d^mid^ CD19^mid^ CD21^low^ CD23^+^ CD43^−^ CXCR5+ IgM^low^ IgD^high^.	Reported missing in fish
Marginal zone B cells	CD1c+ CD19+ CD20+ CD21+ CD27+ FCRL3 + TACI+	CD45R^+^ CD19^+^ CD21^+^ CD27^+^ CD40^+^.	Not reported in fish
Plasmablasts	BCMA+ CD19^low^ CD27^high^ CD38^+^ CD93^+^ CD138^−^	CD45R^low^ CD19^+^ CD27^high^ CD38^+^ CD138^+^	[34] (blimp or pax5)
Plasma cells	BCMA^+^ BLIMP1^+^ CD19^low^ CD20^low^ CD27^high^ CD38^high^ CD138^+^ CXCR4^+^	CD45R^low^ BLIMP1^+^ CD19^−^ CD27^high^, CD38^low^ CXCR4^high^ CD138^+^.	[34] BLIMP1^+^

**Table 2:** Mouse versus human Bregs.

Class	Type of Bregs	Human	Mice
Nomenclature related to classical B2 cells	Immature Bregs	CD19^+^CD24^hi^CD38^hi^ [44]	-
	T2-MZP	-	CD19^+^ CD21^hi^ CD23^hi^ CD24^hi^
	MZ cells	-	CD19^+^CD21^hi^ CD23^−^
	Plasma blasts	CD27^int^CD38^hi^ [42]	CD138^+^CD44^hi^ [42]
	Plasma cells	-	CD138^hi^IgM^+^TACI^+^CXCR4^+^CD1d^hi^Tim1^int^
	Br1 cells	CD19^+^ CD25^hi^ CD71^hi^	-
	Tim1 B cells	-	Tim1^+^ CD19^+^
	B10	CD19^+^CD24^hi^CD27^+^ [53]	CD19^+^ CD5^+^ CD1d^hi^ [54]

**Table 3:** Feasibility of Breg models based on known evolutionary patterns.

Assumption 1	Assumption 2	Model	Homology	Convergence	Concerted
Transient B cell stages found in sharks	Immature Bregs exist in sharks	SS	Incompatible	Incompatible	Incompatible
		SM	Compatible	Incompatible	Incompatible
		MM	Compatible	Incompatible	Incompatible
	Immature Bregs do not exist in sharks	SS	Incompatible	Incompatible	Incompatible
		SM	Compatible	Incompatible	Incompatible
		MM	Compatible	Incompatible	Incompatible
Transient B cell stages found in sharks	Immature Bregs exist in sharks	SS	Incompatible	Incompatible	Incompatible
		SM	Compatible	Incompatible	Incompatible
		MM	Incompatible	Incompatible	Incompatible
	Immature Bregs do not exist in sharks	SS	Incompatible	Incompatible	Incompatible
		SM	Compatible	Incompatible	Incompatible
		MM	Incompatible	Incompatible	Incompatible

## Data Availability

Data available upon request.
